# Placebo analgesia induced by verbal suggestion in the context of experimentally induced fear and anxiety

**DOI:** 10.1371/journal.pone.0222805

**Published:** 2019-09-24

**Authors:** Karolina Świder, Przemysław Bąbel, Eligiusz Wronka, Clementina M. van Rijn, Joukje M. Oosterman

**Affiliations:** 1 Donders Institute for Brain, Cognition & Behaviour, Radboud University, Nijmegen, The Netherlands; 2 Institute of Psychology, Pain Research Group, Jagiellonian University, Kraków, Poland; 3 Institute of Psychology, Jagiellonian University, Kraków, Poland; Leiden University, NETHERLANDS

## Abstract

The role of state anxiety and state fear in placebo effects is still to be determined. We aimed to investigate the effect of fear of movement-related pain (FMRP) and contextual pain related anxiety (CPRA) on the magnitude of placebo analgesia induced by verbal suggestion. Fifty-six female participants completed a modified voluntary joystick movement paradigm (VJMP) where half participated in a predictable pain condition (PC), in which one of the joystick movements is always followed by pain and the other movement is never followed by pain, and half in an unpredictable pain condition (UC), in which pain was delivered unpredictably. By varying the level of pain predictability, FMRP and CPRA were induced in PC and UC respectively. Colour stimuli were presented at the beginning of each trail. Half of the participants were verbally informed that the green or red colour indicated less painful stimuli (experimental groups), the other half did not receive any suggestion (control groups). We measured self-reported pain intensity, expectancy of pain intensity (PC only), pain related fear and anxiety (eyeblink startle response and self-ratings) and avoidance behaviour (movement-onset latency and duration). The results indicate that the placebo effect was successfully induced in both experimental conditions. In the PC, the placebo effect was predicted by expectancy. Despite the fact that FMRP and CPRA were successfully induced, no difference was found in the magnitude of the placebo effect between PC and UC. Concluding, we did not find a divergent effect of fear and anxiety on placebo analgesia.

## 1. Introduction

There are only a few studies that investigated negative emotional states (anxiety and fear) in a context of placebo analgesia [1–6]. Anxiety is a person’s current level of aversive emotional state modulated by situational factors [7] that entails an appraisal of uncertain or uncontrollable threat [8,9], whereas fear is an aversive reaction elicited by the perception of a specific threat stimulus, whether conditioned or not [8]. In general placebo was shown to affect pain by reducing negative emotional states [1,3,5,6]. Nevertheless, in most previous studies it is unclear whether anxiety or fear was measured because state anxiety and state fear should be elicited by the threat of shock [10] and fear-conditioning paradigm [8], respectively. Furthermore, some authors use either ‘fear’ or ‘anxiety’ [3,4] as synonyms rather than different psychological phenomena. As a result, it is not clear what actually influences placebo effects: fear, anxiety, or both. To address this question there is a need to experimentally differentiate between both emotions.

The voluntary joystick movement paradigm (VJMP) developed by Meulders et al. [11–14] offers a good way to experimentally induce state fear and state anxiety. The VJMP is a pain-relevant fear-conditioning paradigm [12] in which extremity movements function as conditioned stimuli (CSs) and painful stimuli as unconditioned stimuli (USs). In this paradigm, fear of movement-related pain (FMRP) and contextual pain related anxiety (CPRA) [8,15] are elicited in two experimental conditions, respectively: a predictable pain condition (PC) and an unpredictable pain condition (UC). In PC, CS (joystick arm movement) are paired (CS+) or not paired (CS-) with painful US (electric shock) which, after a few repetitions, results in a more fearful response (conditioned response) to the movement that is always followed by a painful stimulus (CS+). In the UC, pain stimuli are delivered in time intervals regardless of the joystick movement. As a result this latter condition induces state anxiety.

Verbal suggestion is one of the main methods of eliciting the placebo effect [16–20]. As such, in the current study we used verbal suggestion to induce the placebo effect in the colour stimuli paradigm [21,22] combined with VJMP. In accordance to previous studies [1,5], it is hypothesised that the use of placebo will reduce negative emotional states and induce placebo analgesia. Moreover, it is predicted that a lower placebo effect would be observed in UC compared to PC, as uncertainty have been associated with increased pain ratings [11]. To check if experimental manipulations induced different emotional states, both subjective and physiological measures were used as a manipulation check.

Placebo effects are suggested to be mediated by conscious expectations [20,23–26], however, the exact role of expectations in the induction of placebo analgesia via verbal suggestion have not been unequivocally confirmed. Only a few placebo studies attempted to measure expectations [1,2,4,20,27–29] and even fewer measured expectations on a trial-by-trial basis [30–32]. Therefore, we aimed to investigate to what extent trial-by-trial expectancy of pain intensity (only in PC) could predict the placebo effect induced by verbal suggestion.

## 2. Materials and methods

### 2.1. Participants

Fifty-nine right-handed, female volunteers (mean age = 24.10 years SD = 4.60, range = 19–49 years) participated in this study. Volunteers were recruited amongst the student population of the Radboud University Nijmegen, the Netherlands, and received remuneration for participation in the study. All participants were healthy, free of pain and not taking any pain medications. Exclusion criteria included pregnancy, neurological, cardiovascular and respiratory diseases, psychiatric disorders and hearing problems. Three participants were excluded from the analyses: two due to technical problems and one due to very slow joystick movements during the experiment and a large age difference (age 49 is considered too high compared to the age range of 19 to 37 of the other participants). Data of the remaining 56 participants were further analysed (mean age = 23.66 years SD = 3.27, range = 19–37 years). They were randomly assigned to one of the four groups: two experimental (Group 1 and 3) and two control groups (Group 2 and 4). Each group consisted of 14 participants (see Table 1).

**Table 1:** Characteristics of the subjects in each experimental group: Means (and standard deviations).

Groups	N	Sex	Age
Predictable experimental—Group 1	14	F	24.21(2.46)
Predictable control—Group 2	14	F	24.14(4.66)
Unpredictable experimental—Group 3	14	F	23.50(3.03)
Unpredictable control—Group 4	14	F	22.79(2.61)

N, number of subjects in each experimental group; F, Female

The participants were informed that they were participating in a study with the objective of determining how fast they could move the joystick when pain stimuli were delivered to their hand. All participants signed a written informed consent prior to the experiment. They were also informed that they could stop participating at any point during the study without giving a reason. The study was approved by the institutional ethics committee of Radboud University in Nijmegen (ECG2012-1301- 005) and by the Research Ethics Committee at the Institute of Psychology of Jagiellonian University in Kraków, Poland.

### 2.2. Stimuli materials

#### 2.2.1. Electrocutaneous stimuli

Electrocutaneous pain stimuli of 200 μs duration were delivered to the volar surface of the dominant forearm through two durable stainless-steel disk electrodes 8 mm in diameter with 30 mm spacing, using a Constant Current High Voltage Stimulator (Digitimer, Welwyn Garden City, England, model DS7AH). In the present study a painful electrocutaneous stimulus was used as an unconditioned stimulus (US). The intensity of painful stimuli were individually set for each of the participants (see 2.3.1 below). In total 96 painful stimuli were delivered during 4 blocks of the experiment (24 stimuli in each block). Successive painful stimuli were separated by approximately 16 s in the PC and the UC. The time between successive painful stimuli varied due to the differences in participants’ reaction times (joystick movements).The participants were not aware that the same intensity stimuli were delivered to their right hand during the whole experiment (except during the calibration phase). The mean intensity of the stimuli calculated for individual intensities for all participants was 17.05 mA, SD = .52 and ranged between 7.5–24 mA.

#### 2.2.2. Colour stimuli and visual stimuli

**Fig 1:**
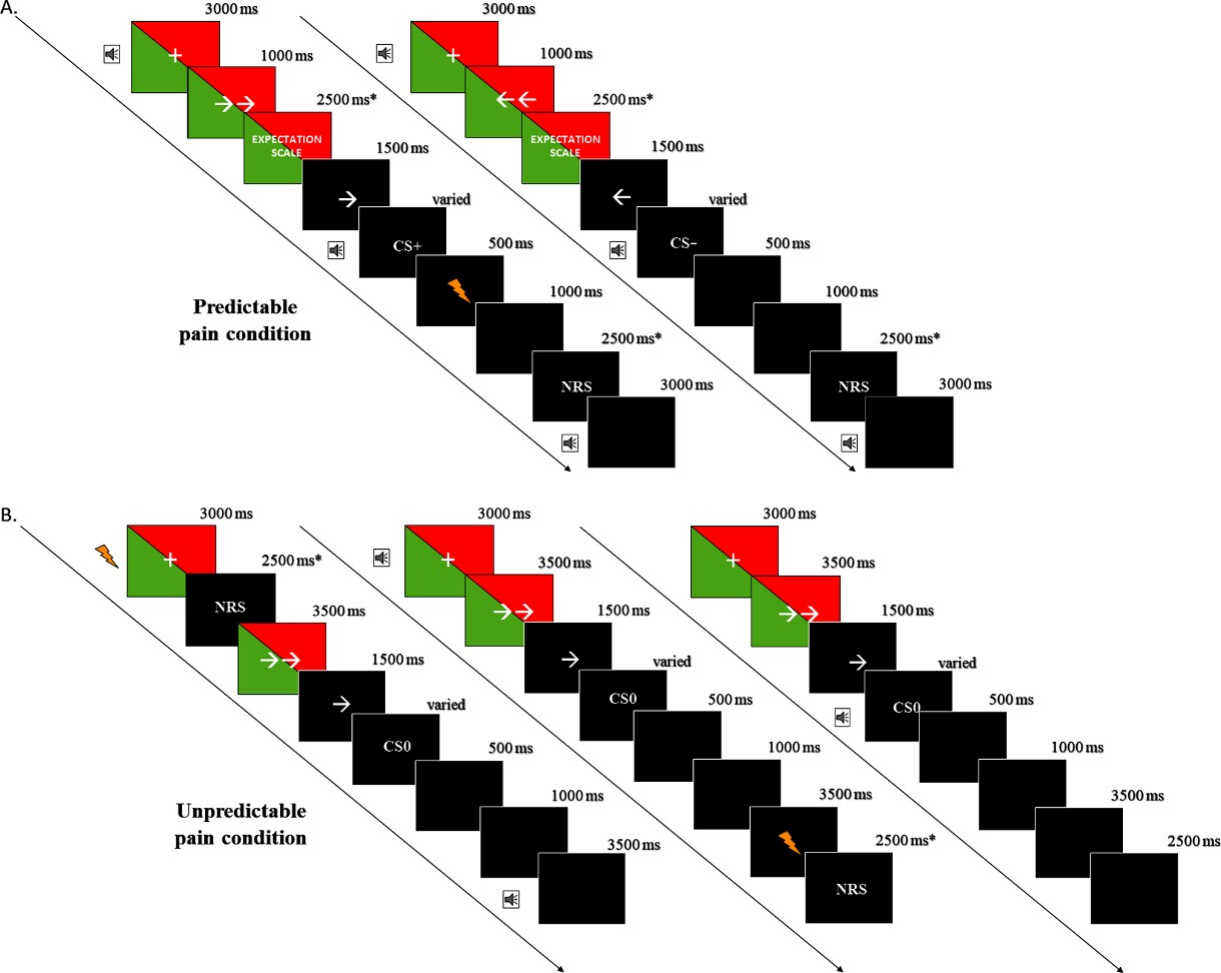
**Design of the testing phase in PC (A) and UC (B).** The timing of the trials differs slightly in accordance to the individual reaction time (movement time). Note: ‘+’ represent*s* fixation cross (FC) when the participants were asked to focus on the FC; ‘NRS’ represents Numerical Rating Scale used to score pain sensations; red and green triangles represent counterbalanced colour stimuli used as placebos; ➔➔ represents the preparation signal anticipating the direction of the movement to be performed (right movement in this case); ➔ represents the starting signal of the movement (right movement in this case); CS+ represents a joystick movement always followed by painful stimuli; CS- represents a joystick movement never followed by painful stimuli in the PC; CSc represents a joystick movement in the UC; speaker represents the startle probe presentation; lightning represents the painful stimuli application. In the PC and UC startle probes were delivered during movements (6 with CS+/CS_right_ and 6 with CS-/CS_left_ in each block of the experiment), in FC presentation (in 2 trials at 2 s; in 2 trials at 2.5 s; in 2 trials at 3 s, in each block) and at the end of the trial (in 2 trials at 2 s after the movement; in 2 trials at 2.5 s after the movement; in 2 trials at 3 s after the movement).

The colour stimuli were full-screen colour slides (red and green) displayed on a computer screen (60 Hz refresh rate) facing the participant at a distance of approximately 50 cm. The red/green colour stimulus was presented for around 6.5 s at the beginning of each trial of the experiment (see Fig 1).

Either the green or red colour acted as a placebo or a control stimulus. The colour stimuli were counterbalanced and the order of their presentation was pseudorandom (no more than 3 consecutive trials containing the same colour stimulus and CS movements). For half of the participants from the experimental groups (Group 1 and 3), the green colour was associated with a placebo-suggestion (information that electrical stimuli following green colour will be less painful) and the red colour was a control stimulus (non-placebo). For the remaining half of the participants in the experimental groups, the meaning of colour stimuli was reversed: green was the control stimulus and red was the placebo stimulus. A total of 192 colour stimuli were presented: 24 red and 24 green in each block of the experiment.

During the experiment two types of visual stimuli were used: two arrows or a single arrow, presented during the colour stimulus presentation (see Fig 1). At the beginning, two arrows (both pointing in the same direction) were presented for 3.5 s and represented a preparation signal anticipating the direction of the movement to be performed. Participants were asked not to move the joystick during this visual stimulus presentation. Afterwards, a single arrow was presented for 1.5 s that always pointed in the same direction as the two arrows. It represented a starting signal for the joystick movement (leftward arrow–left movement; rightward arrow–right movement; 32x32 pixels).

#### 2.2.3. Joystick movements

During the testing phase of the experiment (see 2.3.4 below) the participants were asked to move a Logitech Attack 3 joystick to the left and to the right and these movements served as conditioned stimuli (CSs). The preparation signal (two arrows) announced the direction of the movements at the beginning of each trial. The participants were asked to perform the movement as soon as the starting signal (a single arrow) disappeared from the centre of the black background screen. The proper joystick movement was precisely specified and the participants learned how to move it in the practice phase of the experiment (see 2.3.3 below).

#### 2.2.4. Startle probe

The startle probe was a burst of white noise with instantaneous rise time (100dBA, 50 ms) presented binaurally by SONY stereo headphones. Startle probes are ideal for collecting a startle blink reflex (startle eyeblink reflex—SBR) [33]. The loudness of the startle probe was measured set using a Sound Pressuremeter *Brüel & Kjær* (Amplifier Type 2610, microphone 4192, Artificial Ear Type 4153; Denmark). Startle probes were delivered during the startle habituation phase and testing phase of the experiment (see 2.3.2 and 2.3.4 below).

### 2.3. Procedure

The present study, analogous to Meulders et al. [11,12,34], consisted of 4 phases: a calibration phase, a startle habituation phase, a practice phase and a testing phase lasting around 6, 5, 5 and 50 minutes, respectively. The time of testing phase of the experiment vary due to the different reaction times (joystick movements) and did not include between blocks brakes. The experiment was performed in a sound-attenuated laboratory room. The participants were seated in a comfortable armchair in front of a computer screen at a distance of roughly 50 cm with both of their hands resting on the desk or with their right hand holding the joystick and their left hand placed near the keyboard to be able to use its buttons when it was necessary to score their pain experience.

An adapted version of the VJMP [11,12,34] was used in order to investigate the placebo effect. As was the case in the studies of Meulders et al. [11,12,34], two conditions of the experiment were incorporated, namely predictable pain (PC; Group 1 and 2) and unpredictable pain (UC; Group 3 and 4) in order to elicit fear of movement-related pain and pain-related anxiety, respectively [11,12,34]. In the PC (in both experimental and control group), one of two possible joystick movements was always followed by a painful stimulus (the direction of this CS+ movement was counterbalanced across the participants). In the UC (in both experimental and control group) pain stimuli were never associated with the joystick movement but were delivered after a specific inter trial interval (ITI) at the beginning or at the end of the trial (see Fig 1B).

The adjustment of the VJMP allowed a placebo manipulation to be implemented. Firstly, the verbal suggestion procedure was introduced to elicit placebo analgesia where colour stimuli served as placebos. Secondly, a control condition was incorporated where no suggestion about the meaning of the colours was provided (control groups). Participants from the experimental groups (Group 1 and 3) were verbally informed about the association between placebo stimuli and less painful stimuli applications. Such information was also provided during the experimental procedure in the form of a description presented on the computer screen. In the control groups no information about the association of placebo stimuli and stimulus intensity was provided (Group 2 and 4). Crucially, the participants were not aware that they received the same intensity of stimuli throughout the experiment. Moreover, in the present study participants underwent only one experimental condition (PC or UC). Due to this fact the procedure did not include two long-lasting, background sounds that were associated with the PC and the UC, as was done in the studies of Meulders et al. [11,12,34].

The four phases of the experiment were slightly different in the PC and the UC (see below). Trial timing was not equal, (some participants took more time to move the joystick).

#### 2.3.1. Calibration phase

The calibration was conducted to select the individual painful stimuli for each participant. During this phase the participants were asked to rate the intensity of each stimulus delivered to their forehand on an 11-point numeric rating scale (NRS) ranging from 0 –“no pain”, to 10 –“the strongest pain ever”. Participants were informed that the experiment was targeting a score of 7 (‘moderate pain’) on the NRS and that 10 on the scale indicated the intensity that they did not want to receive anymore. To set these individualized painful stimulus, the participants received one series of electrical stimuli of increasing intensity (1 mA per step, starting from 0) to the value at which an NRS score of 7 was obtained. Next, the intensity of electrical stimuli was decreased (0.5 per step) to an NRS score of 6 and next increased until again a value of 7 on the NRS was obtained. The average of the three values of the individually scored stimulus intensity that was rated as a 7 on the NRS was used as painful stimulus during the experiment. The inter-trial interval (ITI) between each of the electrical stimulus applications was 10 s. During the calibration phase, participants were instructed to notify the experimenter when they did not want to proceed to receiving stimulations of higher intensity.

#### 2.3.2. Startle habituation phase

A startle habituation phase was performed to prevent potential confounds in the data that could be caused by an increased reaction to first startle probes. We presented 1 startle probe during 12 consecutive trials. Each trial lasted for 24 s. The timing of the startle probe presentation was different in each trial (5, 10, 15 and 20 s) and was randomized over trials for each participant (see [11]). During the startle habituation phase no painful stimuli were delivered and participants did not perform joystick movements.

#### 2.3.3. Practice phase

In this phase participants had to perform 14 correct joystick movements. The main task was to move the joystick (7 rightward and 7 leftward movements performed in a pseudorandom order) as quickly as possible when a starting signal (left or rightward arrow) disappeared from the computer screen. Moreover, during this phase, the participants received detailed feedback to learn how to operate the joystick accurately. They monitored the joystick movement via a cursor displayed on the computer screen with the target regions visible. The target region was a big white square presented on the computer screen. The participants were informed if their movement was performed correctly (text information: “Good” appeared at the computer screen). Additional error information was provided in the case of: wrong movement direction (text information: “Wrong direction” if the movement was performed in the opposite direction to which the single arrow was pointing); incomplete movement (text information: “Wrong movement” if the cursor did not reach the target region); slow movement (text information “Too slow”) if the movement lasted more than 3s. Trials with an incorrect movement were repeated until a correct movement was performed. Additionally, in the PC (Group 1 and 2) in 7 of 14 trials pain stimuli were delivered after one of the movements (right or left, counterbalanced across participants). Due to this, the participants learned the association between a specific movement and pain stimulus application. In the UC (Group 3 and 4) no pain stimuli were delivered during this phase.

#### 2.3.4. Testing phase

During this phase in both the PC (Group 1 and 2) and the UC (Group 3 and 4), the colour stimuli, electric shocks and startle probes were presented. The target region, cursor and error messages were not displayed anymore. Before the testing phase, the position of the electrode was changed to prevent stimulation of the same skin area–the electrode was moved slightly away from the palm towards the elbow by approximately 2 cm. In both conditions of the experiment the participants’ task was to move the joystick as fast and as accurately as possible after the presentation of the starting signal (single arrow) and score their pain sensation when the NRS scale appeared on the computer screen.

The experimental phase consisted of 4 blocks of 48 trials each (24 rightward and 24 leftward movements each). Trial timing and trial types of the PC and UC are presented in Fig 1. Each trial started with the colour stimulus presentation. In each block there were 24 trials in which the colour green and 24 trials in which the colour red was presented. The trial order was pseudorandomized. There were no more than 3 consecutive trials of the same joystick movement, no more than 3 consecutive trials using the same colour stimulus and no more than 2 consecutive trials with the startle probe presentation. During the colour stimulus presentation, the white fixation cross (FC) and preparation signal (two arrows) were also presented (see Fig 1).

The experimental phase differed slightly between the PC and UC (see Fig 1). In the PC, one type of movement was always followed by a painful stimulus (CS+ movement). The movement site associated with pain stimuli was counterbalanced across the participants. One half of participants received painful stimuli after the rightward and the other half after the leftward movement. In the UC, pain stimuli were never delivered after joystick movement but were delivered at the beginning of the trial (during FC) or at the end of the trial, 3–5 s after the movement (see Fig 1). In the PC, in contrast to the UC, the participants were asked to rate their expectances about the intensity of the stimulus they were about to receive (see 2.4.2. for details).

In the PC, the startle probe was presented at one of the following moments during the trial: during the fixation cross and colour stimulus presentation (6 trials in one block); during the movement followed by the pain application (CS+; 6 trials in one block); during the movement not followed by the pain application (CS-; 6 trials in one block); or during the break in between trials (6 trials in one block) (see Fig 1). In the UC, the startle probe was presented during the fixation cross and colour stimuli presentation (6 trials in one block) when the pain stimuli was delivered at the end of the trial; during the right (CS_right_) and the left movements (CS_left_) (6 trials in one block for one movement type); or during the break in between trials (6 trials in one block) when the pain stimuli were delivered with the FC (see Fig 1).

### 2.4. Measurements

#### 2.4.1. Pain intensity

In the PC and in the UC of the experiment, the participants rated their pain sensation after each electric stimulus application on an 11-point Likert scale ranging from 0 –“no pain” to 10 –“the strongest pain ever” (the intensity that they don’t want to receive anymore). The scale was presented for 2.5 sec after the pain stimulus application (see Fig 1).

#### 2.4.2. Expectancy of the pain stimuli intensity

In all trials of the PC participants answered the question “How painful do you expect the stimulus will be after performing the left/right movement” and scored their sensation on a 11-point Likert scale with ranging from 0 –“no pain” to 10 –“the strongest pain ever”. The scale was presented for 2.5 s, during the colour stimuli and the preparation signal (two arrows) presentation (see Fig 1). It should be mentioned that in the UC expectancy ratings were not collected due to the characteristics of the procedure–unpredictability of the stimulus application.

#### 2.4.3. Defensive conditioning responses

Regarding retrospective fear of movement-related pain during the CSs, for both conditions of the experiment, after each block the participants answered the question “How fearful were you to perform the right/left movement”. The answer was scored on an 11-point Likert scale ranging from 0 –“not fearful at all” to 10 –“the worst fear I can imagine”. They were asked about fear of performing the CS+ movement and also the CS- movement in the PC and about fear of performing the CS to right (CS_right_) and left (CS_left_) side in the UC.

Regarding response latency and duration, the start region and target region of the joystick movement were identified by a small and a large circle presented on the computer screen, respectively. These circles were visible only during the practice phase. Response latency (movement initiation latency time) was defined as the time to start the joystick movement in accordance to the direction pointed by the arrow stimulus. Response latency (T1) was technically defined as the time from the moment of disappearance of the arrow until participants left the start region (small circle). The small circle was placed in the middle of the screen (17” size and resolution 1920:1080). The radius of the small circle was r = 52 pixels. Response duration (T2) was defined as the time from leaving the small circle (start region) until leaving the large circle (target region), which indicated that participants had successfully completed a movement. The radius of the large circle was r = 370 pixels. A correct movement was defined as reaching the left/right hemisphere of the large circle.

For both T1 and T2 reaction times under 0.2 s, above 3 s and larger than 2.5 SD from the truncated mean of its corresponding cell in the experimental design (the mean for all trials of the participants in the given experimental group) were discarded from the analysis in accordance with Lachaud et al. [35]. The removed data are: (1) Group 1: 9.7% of trials of T1 and 6.69% of trial of T2; (2) Group 2: 20.31% of trials of T1 and 13,54% of trial of T2; (3) Group 3: 13.16% of trials of T1 and 6.84% of trial of T2; (4) Group 4: 11,94% of trials of T1 and 7.44% of trial of T2. From the remaining data, the mean reaction time for each participant was calculated for each CS movement in each experimental group (CS+, CS-, CS_right_ and CS_left_).

#### 2.4.4. Eye blink startle modulation

The eye blink startle responses collected during the CS movements were used as psychophysiological correlates of FMRP and CRPA, indexed by ITI startle responses. The orbicularis oculi electromyography activity (EMG) was recorded with four electrodes: two attached to the external canthi of both eyes (monitoring horizontal eye movement) and one above and one below the left eye (monitoring vertical movement, i.e. blinks). The EMG ground electrode was placed on the nose. Signals were recorded with a Brain Vision Recorder (Brain Products, Munich, Germany) using a 150 Hz low-pass filter, with a time constant of 10 s (0,016 Hz) and a 500 Hz sampling frequency. The signal from the complete experiment was digitized at 1000 Hz and MatLab R2013a software (student edition) was used to perform postprocessing. First, EEG data were loaded using EEGLAB Toolbox (Version 14.0.0). Secondly, data were manipulated according to [33] by performing rectification, variable-weight FIR filtering (101 coefficients, low-pass cut-off frequency 40Hz), and vertical movement electrodes signal summation. The summed signal was then epoched about the startle probe marker (marking the 0 ms origin) in the range -200 ms to 1000 ms. Analogously to Meulders et al. [11,12], the summed signal was baselined by averaging the signal in the range -20 ms to 0 ms. The peak amplitudes were defined as the maximum of the response curve within 21–175 ms after the startle probe onset. Startle waveforms (raw signal for all four electrodes) were visually inspected off-line in order to exclude technical abnormalities and artefacts. Participants with no observed blink responses in either CS or ITI were excluded from the data set. Averages were calculated for responding during CS movements (CS+, CS-, CS_right_ and CS_left_) and ITI (mean startle amplitude collected during FC and during the break in between trials) separately for both the PC and the UC. Data collected from 20 participants from the PC and 20 participants from the UC were used in further statistical analysis.

## 3. Statistical analysis

In order to test our hypotheses, data from the testing phase of the study were analysed. The NRS pain intensity ratings were averaged from blocks 1 to 4 in the PC and the UC. Two dependent variables were analysed: (1) the mean pain intensity of placebo- and non-placebo-associated pain ratings and (2) the mean difference between placebo- and non-placebo-associated pain ratings in experimental groups; and the mean difference between red- and green-associated pain ratings in control groups. Note that in control conditions placebo and non-placebo stimuli refer to mean ratings of green- and red- associated stimuli, respectively. Similarly, the NRS expectancy of the pain intensity ratings, from blocks 1 to 4 in the PC, were averaged, and dependent variables were analysed: (1) the mean placebo- and non-placebo-associated expectancy of pain intensity ratings^3^ and (2) the mean difference between placebo- and non-placebo-associated expectancy of pain intensity ratings in the experimental group; and the mean difference between red- and green-associated expectancy of pain intensity ratings in the control group.

### 3.1. Analysis of variance

Separate repeated measures ANOVAs were conducted for each dependent variable: NRS pain intensity ratings and NRS expectancy of pain intensity. A repeated measures ANOVA for NRS pain intensity ratings, with Condition (predictable and unpredictable) and Group (experimental and control) as between-subjects factors and Placebo (placebo and non-placebo stimuli) as a within-subjects factor was performed. For NRS expectancy of pain intensity scores, the repeated measures ANOVA with between-subjects factor Group (experimental and control) and within-subjects factor Placebo (placebo and non-placebo stimuli) was computed.

In order to test whether verbal suggestion was effective, the repeated measures ANOVA was followed by within-group planned comparison tests: (1) placebo- versus non-placebo-associated NRS ratings in the experimental group, and (2) red-control versus green-control NRS ratings in the control group. In the next step of the analyses, in order to investigate whether placebo analgesia was elicited, between-group planned comparison tests were performed on the difference in pain intensity ratings between non-placebo- and placebo-associated stimuli from the experimental groups compared with the difference in pain intensity ratings between red-control and green-control stimuli from the control groups.

In order to test whether verbal suggestion elicited differences in participants’ expectancy (only PC), within-group planned comparison tests were performed for the expected pain intensity scores: (1) placebo- versus non-placebo-associated expectancy of pain intensity scores in the experimental group, and (2) red-control versus green-control expectancy of pain intensity scores in the control group.

### 3.2. Regression analysis

Linear regression analysis was performed for the PC to determine the degree to which the placebo analgesia was predicted by the expected pain intensity. To do this, the mean difference in pain intensity ratings between placebo- and non-placebo-associated stimuli was set as the dependent variable, while the mean differences in the expected pain intensity scores between placebo- and non-placebo-associated stimuli served as an independent variable.

### 3.3. Defensive conditioning responses–manipulation check

In order to investigate if CS+ movements in the PC elicit elevated defensive conditioning responses, for (1) self-reported fear, (2) response latencies (T1), and (3) response duration (T2), repeated measure ANOVAs with Condition (predictable and unpredictable) as a between-subjects factor and Movement Type (CS+/right, CS-/left) as a within-subjects factor were performed. CS+ and CS- refer to predictable condition and CS_right_ and CS_left_ refer to the unpredictable condition. The F-tests were followed by planned comparisons in order to compare the magnitudes of self-reported fear between each movement type (CS+/right, CS-/left) for both experimental conditions (PC and UC).

Additionally, the startle modulation data (eye blink amplitudes) were analysed with a repeated measure ANOVA with Condition (predictable and unpredictable) as a between-subjects factor and Startle Type (CS+/right, CS-/left and ITI)^4^ as a within-subjects factor. Next, F-tests were followed by the post-hoc pairwise comparison with Bonferroni correction in order to compare the magnitude of the three types of startle amplitudes.

## 4. Results

The descriptive statistics for pain intensity ratings and expectancy of the pain intensity are presented in Table 2.

**Table 2:** Descriptive statistics for pain intensity ratings, expectancy of pain intensity score.

Variables	Stimulus	Group 1 Predictable experimental	Group 2 ^b^ Predictable control	Group 3 Unpredictable experimental	Group 4^b^ Unpredictable control
Pain intensity	Placebo	4.48±1.93	3.65±1.44	3.87±1.26	3.85±.79
	Non-placebo	4.95±1.91	3.69±1.42	4.10±1.16	3.84±.74
	Difference^a^	.46± .64	.04±.12	.22±.41	-.01±.14
Expectancy	Placebo	3.61± 1.85	3.26±1.54		
	Non-placebo	4.96±1.49	3.38±1.61		
	Difference^a^	1.35±1.35	.13±.25		

^a^Difference between NRS ratings of non-placebo- or placebo-associated stimuli;

^b^In the control group both stimuli were set up at the same level of intensity, therefore the difference presented here can be considered as a difference between red- and green-associated NRS ratings.

All results of the repeated measures ANOVA on the NRS ratings are presented in Table 3. The repeated measures ANOVA of the NRS ratings revealed a statistically significant main effect of Placebo (*F*_(1,52)_ = 11.62, *p* < .001, *η*^2^ = .03) and a statistically significant interaction of Placebo × Group (*F*_(1,52)_ = 9.85, *p* = .003, *η*^2^ = .03).

**Table 3:** The results of repeated measures ANOVA on the pain intensity ratings.

**Main effects and interactions**	**F**	***df***	***P***	*η*^***2***^
Placebo	11.62	1,52	< .001	.03
Group	2.58	1,52	.12	.37
Condition	.56	1,52	.45	.00
Placebo × Condition	1.90	1,52	.174	.01
Placebo × Group	9.85	1,52	.003	.03
Placebo × Group × Condition	.881	1,52	.352	.00
**Within-group planned comparisons**	**F**	***df***	***P***	*η*^***2***^_***p***_
Experimental groups (placebo-associated stimuli vs non-placebo-associated stimuli)	21.44	1,52	< .001	.29
Control groups (green-control stimuli vs red-control stimuli)	.04	1,52	.85	.00
**Between-group planned comparisons**	**F**	***df***	***P***	*η*^***2***^
The mean difference in NRS pain scores between placebo- and non-placebo stimuli from the experimental groups vs the mean difference between two control (red and green) stimuli from the control groups.	9.71	1.54	.003	.15

Within-group planned comparison tests on the NRS ratings (see Table 3) revealed a statistically significant difference in the experimental groups (Group 1 and Group 3, mean difference .34 ± .07), indicating that pain stimuli associated with the placebo suggestions were rated as less painful than the non-placebo ones. No such effect was observed in the control groups (Group 2 and 4, mean difference .01 ± .07).

**Fig 2:**
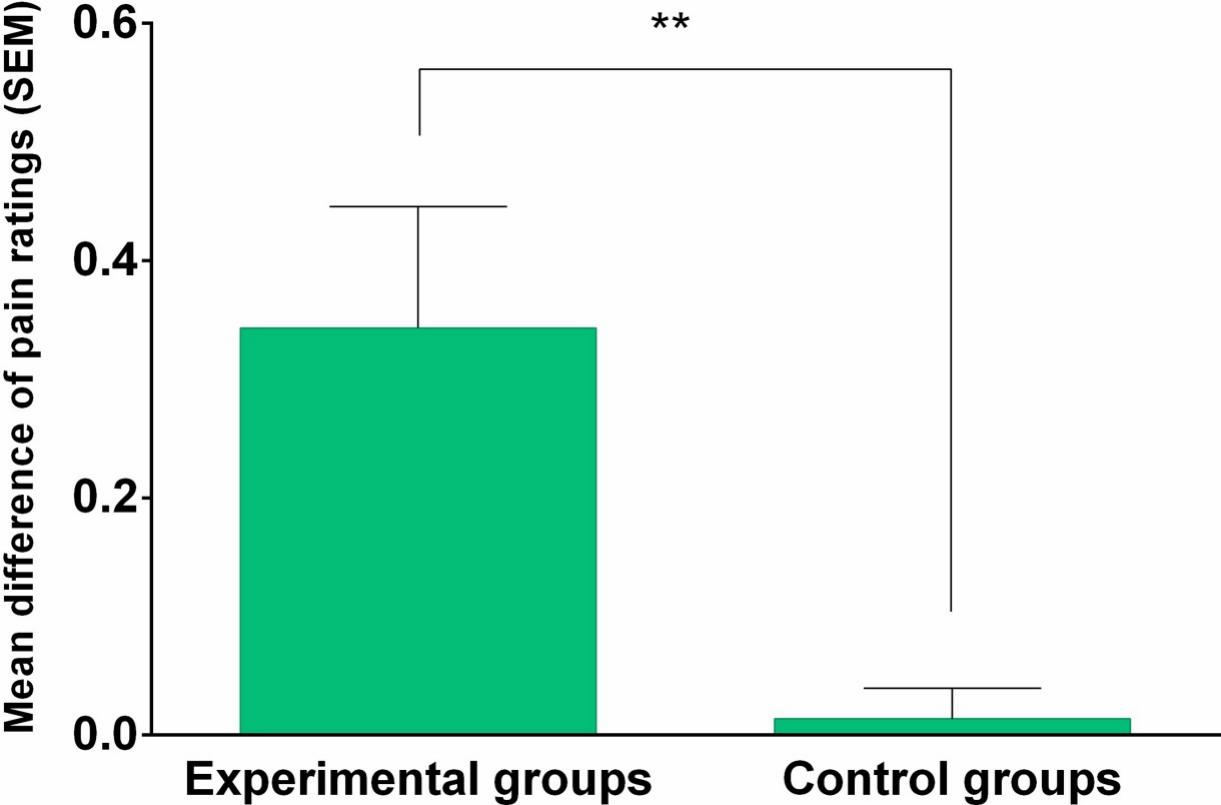
Placebo effect. The difference in pain intensity ratings between non-placebo and placebo-associated stimuli from the experimental groups and the difference in pain intensity ratings between red-control and green-control stimuli from the control groups.

Between-group planned comparison tests on the difference in pain intensity ratings between non-placebo- and placebo-associated stimuli from the experimental groups (mean .34 ± .54) compared with the difference in pain intensity ratings between red-control and green-control stimuli from the control groups (mean .01 ± .13) was performed. It revealed a statistically significant effect (see Table 3 and Fig 2), indicating that placebo analgesia was induced by verbal suggestion in the experimental groups.

**Fig 3:**
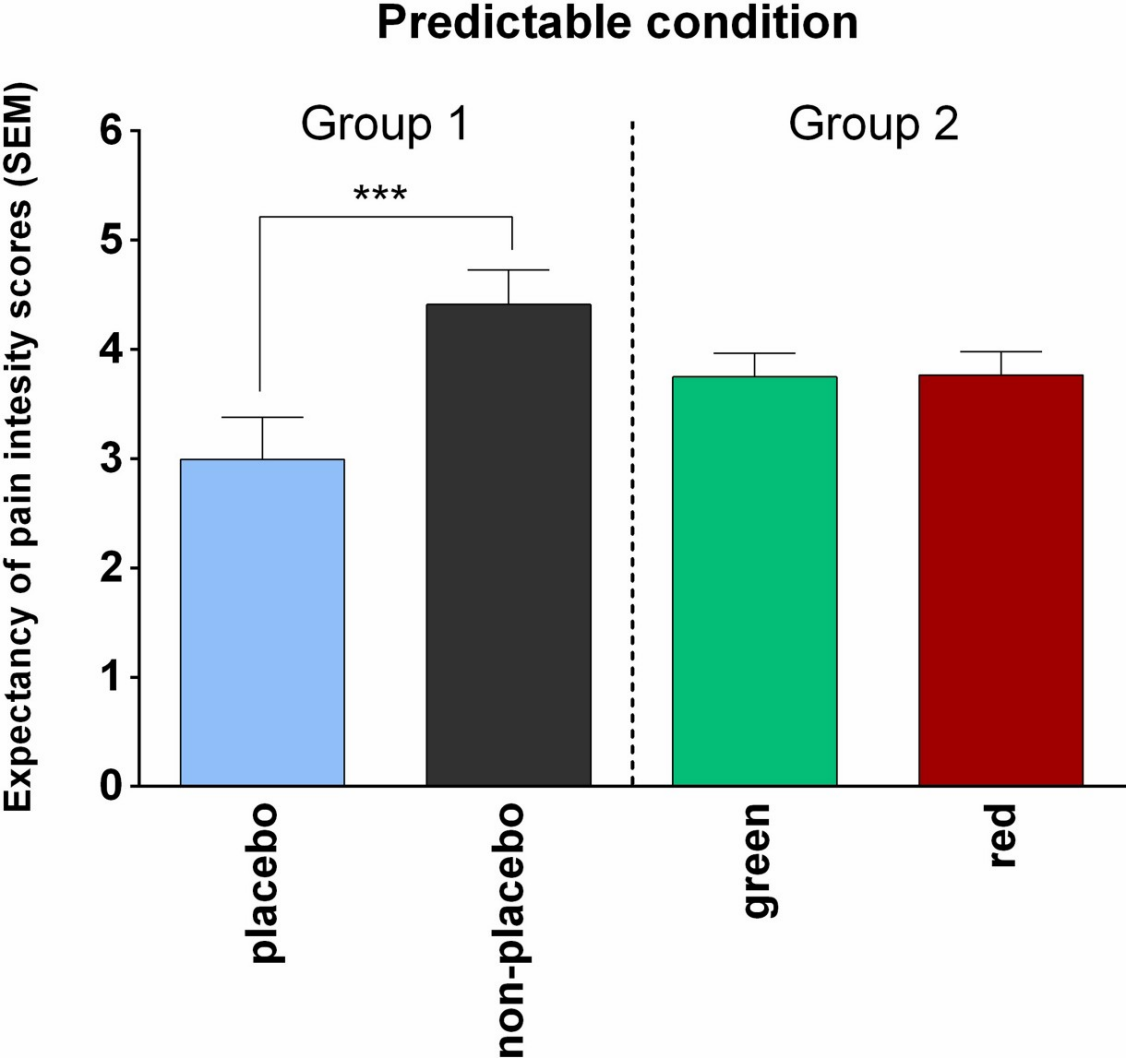
Expectancy of pain intensity scores in the predictable condition. In experimental group (only PC), verbal suggestion about the analgesia had an effect on expectancy of pain intensity.

All results of the repeated measures ANOVA on the expectancy of pain intensity scores are presented in Table 4, showing a statistically significant main effect of the Placebo (*F*_(1,26)_ = 15.46, *p* < .001, *η*^2^ = .30) and a significant interaction effect of Placebo × Group (*F*_(1,26)_ = 10.653, *p* = .003, *η*^2^ = .20). Within-group planned comparison (see Table 4 and Fig 3) on non-placebo- versus placebo-associated expectancy of pain intensity scores revealed a statistically significant difference for the experimental group (Group 1, mean difference 1.34 ± .26), indicating that verbal suggestion had an effect on expectancy of pain intensity. By contrast, no statistically significant difference was found between two control stimuli (red and green) in the control group (Group 2, mean difference .13 ± .26).

**Table 4:** The results of repeated measures ANOVA on the expectancy of pain intensity.

**Main effects and interactions**	**F**	***df***	***P***	*η*^***2***^
Placebo	15.46	1,26	< .001	.30
Group	2.69	1,26	.113	09
Placebo × Group	10.65	1,26	.003	.20
**Within group planned comparisons**	**F**	***df***	***P***	*η*^***2***^_***p***_
Experimental groups (placebo-associated stimuli vs non-placebo-associated stimuli)	25.88	1,26	< .001	.49
Control groups (green-control stimuli vs red-control stimuli)	.64	1,26	.22	.01

A simple linear regression was performed to predict the magnitude of the placebo effect based on expectancy of pain intensity scores. It revealed a significant regression equation (*F*_(1,26)_ = 8.85, *p* = .006) with *R*^*2*^ of .254. A positive correlation was found between the placebo effect and the expectancy of pain intensity scores (r = .504, p = .003), and the regression model predicted 25.4% of the variance.

**Fig 4:**
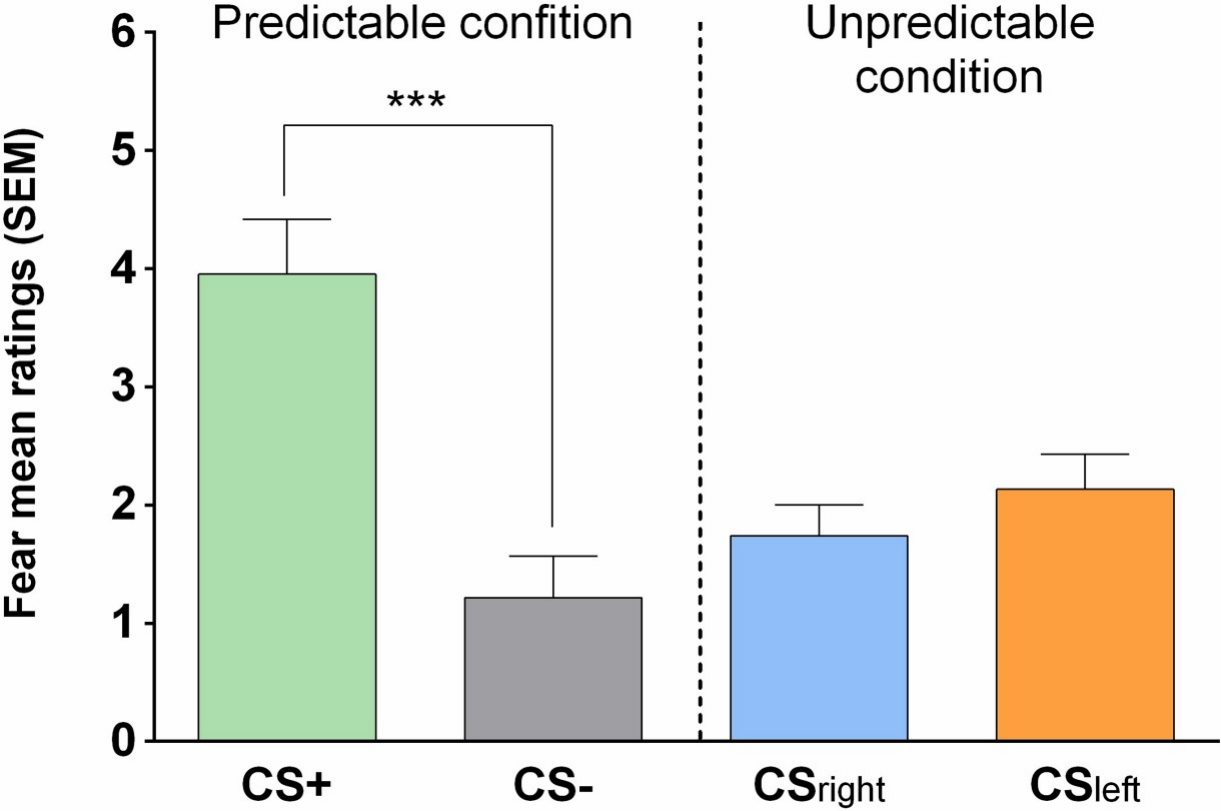
Fear ratings associated with the type of movement. In the PC, the level of fear was the highest for the movement associated with pain application (CS+) compared to the movement never associated with pain application (CS-). In the UC, no difference was found for fear level associated with the CS_right_ and CS_left_ movements.

Results of the repeated measures ANOVA performed on self-reported fear ratings revealed statistically significant main effects of Condition (*F*_(1,54)_ = 50.51, *p* < .0001, *η*^2^ = .38) and Movement Type (*F*_(1,54)_ = 28.36, *p* < .001, *η*^2^ = .21). Moreover, a statistically significant interaction of Condition × Movement Type (*F*_(1,54)_ = 9.85, *p* = .003, *η*_*p*_^2^ = .03) was also observed. Planned comparison tests revealed that in the PC, participants were more afraid of CS+ movement than CS- movements (*F*_(1,54)_ = 77.29, *p* < .001, *η*_*p*_^2^ = .09). No difference between self-reported fear of CS_right_ and CS_left_ in the UC was found (*F*_(1,54)_ = 1.58, *p* > .05, *η*_*p*_^2^ = .03) (see Fig 4).

The repeated measures ANOVA performed on response latencies (T1) revealed a statistically significant main effect of Condition (*F*_(1,54)_ = 4.77, *p* = .03, *η*^2^ = .08) indicating that participants were slower in initiating a movement in the PC compared to the UC (mean .47 ± .09 and .43 ± .07, respectively). No statistically significant main effect of Movement Type (*F*_(1,54)_ = 2.59, *p* > .05, *η*^2^ = .05) nor an interaction of Condition × Movement Type (*F*_(1,54)_ = .19, *p* > .05, *η*^2^ = .003) was found.

Results of the repeated measures ANOVA performed on response duration (T2) revealed a statistically significant main effect of Condition (*F*_(1,54)_ = 4.43, *p* = .04, *η*^2^ = .08), indicating slower joystick movements in the PC compared to the UC (mean .54 ± .10 and .49 ± .08, respectively). No statistically significant main effect of Movement Type (*F*_(1,54)_ = .05, *p* > .05, *η*^2^ = .001) nor an interaction effect of Condition × Movement Type (*F*_(1,54)_ = 1.67, *p* > .05, *η*^2^ = .03) was found. The obtained results for T1 and T2 suggest no differences between movement types.

An additional repeated measures ANOVA performed on the startle response amplitude revealed significant main effects of Condition (*F*_(2,76)_ = 4.92, *p* = .03, *η*^2^ = .12) and Startle Type (*F*_(2,76)_ = 4.83, *p* = .01, *η*^2^ = .11). However, no significant Startle Type × Condition interaction was found, indicating that the startle amplitude in response to the ITI and the different CSs movements did not differ significantly for both experimental conditions (*F*_(2,76)_ = 1.32, *p* = .27, *η*^2^ = .03). Startle reflexes in the PC were higher than in the UC (360.71 ± 162.06 and 282.69 ± 124.09, respectively). Pairwise comparisons with Bonferroni correction performed for the three types of startle response revealed that CS+ and CS- startle response do not differ (*p* >. 05); however, both have higher amplitudes compared to the ITI startle amplitude (*p* < .05 for comparison of both CS+ and CS- with the ITI startle responses).

## 5. Discussion

In the present study, we examined using VJMP [12] if FMRP and CPRA have different effects on the magnitude of placebo analgesia induced by verbal suggestion. Moreover, an attempt was made to investigate if induced placebo analgesia can be predicted by expectancy of pain intensity (only PC). The results were largely in line with the predictions.

### 5.1. Placebo effect and negative emotional states

Placebo analgesia was induced in both experimental conditions. This result is coherent with previous findings showing that verbal suggestion is sufficient to induce the placebo effect [16–20].

Aside from the studies of Meulders et al. [11,12,34], only a few studies focused on the divergent effects of fear/anxiety on pain sensitivity [10,36]. To our knowledge no such attempt was made in the context of placebo effects. In contrast to our expectations, we did not observe any divergent effects of FMRP and CPRA on the magnitude of the placebo effect. This result contradicts our hypothesis that a weaker placebo effect would be elicited in the UC. Our results suggest that state fear and state anxiety influence the placebo effect in the same manner. However, this conclusion needs further investigation where control group with neither fear nor anxiety elicitation is included in the study design. Such control group is difficult to create in the modified joystick paradigm. Therefore, this issue should be resolved using a distinct paradigm.

However, we cannot exclude that differences in the magnitude of the placebo effect induced in the context of state fear and anxiety exist. Such differences may be visible with different intensities of particular emotional states. The emotions level can be manipulated by changing the duration, intensity or the location of pain stimuli [37]. However, in the present study neither fear nor anxiety levels were manipulated. Therefore, future investigation is needed.

Moreover, the measurement of the placebo effect (via verbal ratings) might not be sufficiently precise and sensitive to capture the influence of FMRP and CPRA. Behavioural measurements of pain have been shown to be less sensitive because they are the end product of all preceding cumulative cognitive operations and are more vulnerable to noise and variability. EEG studies could be more effective in capturing the differences in the magnitude of the placebo effect in both PC and UC, as previous studies showed that placebo suggestions decreased brain activity in the regions related to anxiety processing [38,39].

### 5.2. Placebo effect and expectancy

The effects of verbal suggestion are considered to be mediated by expectancies [20,23–26]. To our knowledge there is only one study in which expectations were found to influence the nocebo effect induced by verbal suggestion [40]. In the present study, the participants expected less pain in relation to placebo stimuli than non-placebo stimuli. Moreover, it was demonstrated that the placebo effect was predicted by expectancy which is in line with the expectancy theory [24,41] and Colloca and Miller’s [23,42] learning model. Specifically, verbal information about pain decrease elicits expectations and modulates pain perception. To the best of our knowledge, this is the first study in which the placebo effect induced by verbal suggestion only (without conditioning nor other interventions) was found to be predicted by expectations. Büchel et al. [43] proposed a theory linking expectancy and the placebo effect. They suggested that “combining top-down prior expectations or predictions of pain (relief) with bottom-up sensory signals at multiple levels of the neural hierarchy” induces placebo analgesia. Moreover, they stressed the role of precision and certainty of the expectancy in eliciting the placebo effect. Based on their work and studies on neurobiological mechanisms of the placebo effect [44,45], it is speculated that modulatory neurotransmitters, such as opioids [46] or dopamine [47,48], might be related to the characterization of expectations induced by verbal suggestions. It is suggested to investigate in the future the EEG correlates of expectations such as contingent negative variation (CSV) that is elicited before an incoming painful stimulation. This central EEG measures were used in the study of Piedimonte et al. [49] where authors used an expectation paradigm with and without conditioning procedure and focused on the expectation in placebo and nocebo effect.

### 5.3. Manipulation check

Results obtained for self-reported fear revealed that the manipulation performed in PC succeeded in eliciting FMRP. Specifically, as we predicted, elevated fear ratings were observed for CS+ movements compared to CS- movements, suggesting that our fear conditioning procedure was effective. This result is in line with the results obtained in other studies where VJMP was used [11,12] and indicates that FMRP can be elicited not only by social learning [50,51] but also by experience.

Analysis performed for the T1 and T2 partially indicates that FMRP and its accompanying avoidance behaviour were induced. In comparison to UC, in PC the participants were more reluctant to initiate movements (T1) and they needed more time to perform the movements (T2). However, in contrary to previous findings of Meulders et al. [11,12], the participants in PC did not show a tendency to avoid CS+ movements more than CS- movements.

As in the present study, Meulders et al. [12] could not provide a conclusive explanation for their findings that T2 for CS+ and CS- movements did not differ. Results obtained for both T1 and T2 might be associated with strict preprocessing of raw data—we discarded high percentages of trials from the analysis (see 2.4.3). The filtering of reaction time data may introduce a bias [35]. Another explanation, which is supported by the amount of discarded data, is that there may have been some procedure apparatus problems. It is highly probable that the joystick movement sensitivity could have been too high (e.g. the radius of the small circle was too small) resulting in a high number of trials below 0.2 s).

The startle response amplitude was shown to be higher in the PC compared to the UC regardless of the type of startle, which suggests the presence of elevated conditioned defensive response in the PC. Moreover, regardless of the experimental condition, CS+/CS_right_ and CS-/CS_left_ startle responses had higher amplitudes than those associated with ITI startle. Startle measures were not elevated more in response to the CS+ than to the CS-, which contradicts previous findings [11,12] and which may possibly be related to the small number of trials collected for each startle.

It is suggested that results obtained for self-reported fear, T1, T2 and startle responses may indicate that in both experimental conditions differences in emotional arousal were present and that FMRP and CFRA were successfully elicited. In accordance with Davis et al. (for review see [52]), the sustained response to temporally uncertain danger is associated with the elicitation of anxiety, one of two primary defensive behavioural states. The second defensive behavioural state is fear, which is defined as a phasic response to imminent threat. Crucially, both responses are shown to be subserved by distinct neural substrates [53]. Another argument supporting this assumption arises from a study in which unpredictability of pain application was shown to elicit CPRA [11–13]. In those studies CPRA was indexed by elevated ITI startle responses in the UC compared to the PC. Therefore, it can be assumed that our procedure used to elicit FMRP and CRPA was effective, even though there was no difference between the CS+ and CS- startle response nor between the UC and PC ITI startle responses.

### 5.4. Limitations

Some limitations of the present study should be acknowledged. First, only healthy female participants were tested. Therefore, the conclusions of this research are applicable to the healthy female population only. Second, as explained before, our joystick settings might have been too sensitive. It is also probable that no differences in the magnitude of the placebo effect between the PC and the UC could be associated with insufficient unpredictability of pain stimuli in the latter. Pain stimuli in the UC could have been associated with ITI startle probe presentation what would have made this condition less unpredictable. It is suggested to use STAI questionnaire at the beginning of the experiment to be able to check if the anxiety level for both groups is the same.

### 5.5. Conclusions

Placebo analgesia was observed in both the PC and the UC; however, no differences were found in the magnitude of the effect between both experimental conditions. For the first time expectancy was shown to predict the placebo effect induced via verbal suggestion in a context of experimentally induced state fear. Finally, some of our manipulation checks (eyeblink startle and self-reported fear) indicate that FMRP and CPRA were successfully elicited in the PC and the UC, respectively. Further research using objective measures, such as EEG, is required to investigate the differential role of fear, anxiety and expectation in the placebo effect induction. Next, it is recommended to collect trial-by-trial fear ratings and investigate if the level of FMRP, as in the case of physical performance and self-reported disability [54–56], may predict the placebo effect. Finally, it is suggested to implement a modification in the experimental design in which different predictability dimensions are used.

### 5.6. Implications

The adjusted VJMP is a promising research tool that opens a new field of research on the placebo effect in the experimental context of maintenance of chronic musculoskeletal pain. Moreover, in the future, the adjusted VJMP will allow to investigate how the reduction of FMRP and CPRA influences the magnitude of the placebo effect (the method of reducing both emotional states can be found in Meulders et al. [13]). Moreover, VJMP may be implemented to test chronic pain patients and compare them with other clinical populations or healthy participants; however, taking into account the preliminary character of the present study, further investigation is needed before the investigation reaches clinical conditions.
